# Effect of Humanizing Mutations on the Stability of the Llama Single-Domain Variable Region

**DOI:** 10.3390/biom11020163

**Published:** 2021-01-26

**Authors:** Miguel A. Soler, Barbara Medagli, Jiewen Wang, Sandra Oloketuyi, Gregor Bajc, He Huang, Sara Fortuna, Ario de Marco

**Affiliations:** 1 CONCEPT Lab, Italian Institute of Technology (IIT), 16152 Genova, Italy; 2 Department of Chemical and Pharmaceutical Sciences, University of Trieste, 34127 Trieste, Italy; bmedagli@units.it (B.M.); s.fortuna@units.it (S.F.); 3 Key Laboratory of Systems Bioengineering (Ministry of Education), School of Chemical Engineering & Technology, Tianjin University, Tianjin 300072, China; wangjw@tju.edu.cn (J.W.); huang@tju.edu.cn (H.H.); 4 Lab of Environmental and Life Sciences, University of Nova Gorica, 5000 Rožna Dolina-Nova Gorica, Slovenia; sandra.folarin.oloketuyi@ung.si; 5 Department of Biology, Biotechnical Faculty, University of Ljubljana, 1000 Ljubljana, Slovenia; gregor.bajc@bf.uni-lj.si

**Keywords:** nanobody framework, modeling, nanobody humanization, CDR grafting

## Abstract

In vivo clinical applications of nanobodies (VHHs) require molecules that induce minimal immunoresponse and therefore possess sequences as similar as possible to the human VH domain. Although the relative sequence variability in llama nanobodies has been used to identify scaffolds with partially humanized signature, the transformation of the *Camelidae* hallmarks in the framework2 still represents a major problem. We assessed a set of mutants in silico and experimentally to elucidate what is the contribution of single residues to the VHH stability and how their combinations affect the mutant nanobody stability. We described at molecular level how the interaction among residues belonging to different structural elements enabled a model llama nanobody (C8WT, isolated from a naïve library) to be functional and maintain its stability, despite the analysis of its primary sequence would classify it as aggregation-prone. Five chimeras formed by grafting CDRs isolated from different nanobodies into C8WT scaffold were successfully expressed as soluble proteins and both tested clones preserved their antigen binding specificity. We identified a nanobody with human hallmarks that seems suitable for humanizing selected camelid VHHs by grafting heterologous CDRs in its scaffold and could serve for the preparation of a synthetic library of human-like single domains.

## 1. Introduction

Variable regions of heavy-chain-only *Camelidae* antibodies (nanobodies, VHH) are commonly selected by panning both immune and pre-immune libraries of recombinant ligands [1,2,3,4]. In vitro selection protocols can be designed to favor the recovery of binders with desired features in terms of affinity, epitope specificity or resistance to chemical and physical conditions [5,6,7]. The potential antigenicity of nanobodies is irrelevant for most of the research and diagnostic applications but, despite the similarities between VHH and human VH (IgG heavy-chain variable region) domains, it remains an issue for their use in vivo. Consequently, there has always been a strong interest in identifying strategies to humanize VHHs without compromising their structural and functional characteristics [8,9].

Synthetic libraries can be designed to yield partially humanized binders, but the process is incomplete since it does not involve the VH hallmarks in the second framework that are responsible for the domain stability [3]. VHHs recovered using sequences originated by animals can be particularly resistant to humanization and in a previous publication, we showed how amino acid point mutations can affect the single-domain stability by inducing both its unfolding and the colloidal aggregation of folded VHHs that expose hydrophobic residues on their surface [10]. On the other hand, several natural VHHs recovered by panning immune and pre-immune libraries share the unexpected particularity of having a human (or hybrid human/camelid) signature rather than the camelid hallmarks [11,12]. Such antibodies are functional and stable despite their unconventional sequence.

It is known that framework residues can interact with loop amino acids and that such interactions have the double effect of neutralizing potential aggregation spots and of imposing structural rigidity to the paratope [13,14,15]. Since such stabilizing effect is due to interactions between specific residues and these can vary among sequences, it is improbable that grafting loop sequences in “optimized” scaffolds would automatically result in functional binders. It can be therefore argued that the stability of the nanobodies with VH hallmarks might be due to particular neutralizing interactions between the hydrophobic “human” residues and other residues of the sequence but there are no available structures of such odd VHHs to infer definitive conclusions.

In this work we have analyzed systematically: (i) what happens when a VHH with canonical llama sequence (the anti-HER2 [human epidermal growth factor receptor 2] A10, described in [16]) is progressively humanized; (ii) the possibility to use a llama single-domain with human VH signature (the anti-FGFR1 [fibroblast growth factor receptor 1] C8, described in [1]) as a universal acceptor frame for grafting the complementarity-determining regions (CDRs) from other nanobodies. The results confirmed that a complete humanization of a typical camelid sequence is difficult, but we demonstrated that a human-like VHH framework represents a promising scaffold on which grafting different CDRs originally present in conventional VHH.

## 2. Materials and Methods

### 2.1. Nanobody Production

The sequences of the CDRs belonging to VHHs specific for different antigens (GFP, HER2, ALFA tag) were grafted on the framework sequence of C8WT (originally, an anti-FGFR1 VHH described in [1]) and the resulting chimeras were obtained as synthetic genes from Twist Bioscience (San Francisco, CA, USA). These were cloned into the pET14-GFP/mClover vectors [16], with the only exception of the anti-GFP construct that was cloned in the pET14-mCherry vector. The resulting constructs are fusions of VHHs (at the N-term) with a fluorescent protein and a C-terminal 6xHis tag and were expressed in SOX bacteria which express recombinant sulfhydryl oxidase in their cytoplasm [17].

Small-scale expression was performed comparing three different culture media: ZYM 5052 autoinduction media, lysogeny broth (LB) and terrific broth (TB). ZYM 5052 was dismissed because of the low yields. The growth conditions used for the LB were as it follows. Bacteria were grown in an orbital shaker (210 rpm) at 37 °C until OD600 was 0.4, then 0.5% (*w*/*v*) arabinose was added, and the temperature lowered at 30 °C. After 40 min, 0.5 mM IPTG was added and the temperature lowered at 20 °C. Bacteria were collected by centrifugation after overnight culture. Bacteria were grown in TB at similar conditions, but 1% (*w*/*v*) arabinose was added when OD600 reached 0.6.

For large-scale expression, inoculated TB media were cultured as above, the collected pellets (5 g) were resuspended in 30 mL of 25 mM Tris-HCl, pH 7.9, 500 mM NaCl, 5% glycerol, 1 mg/mL lysozyme, 1 mM PMSF, 0.01 mg/mL DNase I, 10 mM MgCl_2_, incubated 1 h under agitation at 4 °C and cells were disrupted using a Dounce homogenizer. The resulting lysate was left further 20 min under agitation at 4 °C before centrifuging it at 4400× *g* for 45 min at 4 °C. Two purification strategies were used. In the first case, the supernatant was recovered and incubated for metal-affinity purification (IMAC) for 1 h at 4 °C with 200 uL of Talon resin (Clontech, Mountain View, CA, USA). The resin was washed first in 4 mL of 25 mM Tris-HCl, pH 7.9, 500 mM NaCl, 5% glycerol and successively in 3 mL of 25 mM Tris-HCl, pH 7.9, 500 mM NaCl, 5% glycerol, 15 mM imidazole. The target proteins were eluted in 4 × 300 μL of 25 mM Tris-HCl, pH 7.9, 500 mM NaCl, 5% glycerol, 150 mM imidazole and the resulting samples were centrifuged at 10,000× *g* for 10 min and then injected in a gel filtration column (Superdex 75 10/30, GE Healthcare, Chicago, IL, USA) equilibrated in phosphate-buffered saline (PBS), pH 7.4, 1 mM DTT. In the alternative protocol, the IMAC step was performed using a 5 mL HiTrap HP column coupled to an ÄKTA system (both GE Healthcare) and the elution fractions were first equilibrated in 50 mM MES, pH 6.0, 20 mM NaCl, 1 mM DTT before undergoing ion exchange chromatography (IEX) using a HiTrap SP HP column (GE Healthcare, Chicago, IL, USA). Proteins were eluted using a linear 20–500 mM NaCl gradient. Protein concentration was determined by Bradford assay.

### 2.2. Stability Tests

Protein samples diluted in PBS (500 μL at a concentration of 200 μg/mL) were filled in a sealed 1.5 mL Eppendorf tube and stored 15 days at −20 °C, 4 °C or 21 °C. At the end of the storage period, samples were centrifuged (10,000× *g*) for 10 min and protein concentration was evaluated by recording the absorbance at 280 nm. The experiment was performed in duplicate.

NanoDSF (Differential Scanning Fluorimetry) has been used for measuring protein thermal unfolding transition midpoint-Tm [°C] using intrinsic tryptophan and tyrosine fluorescence at the emission wavelengths of 330 nm and 350 nm. Measurements were performed in a Prometheus NT.48 instrument (NanoTemper, Munich, Germany) loading 10 μL of sample in each capillary, samples were subjected to a temperature ramp of 1 °C/min from 20 °C to 95 °C and data were analyzed automatically by the device software.

### 2.3. Surface Plasmon Resonance (SRP) Experiments

The VHH binding affinity was evaluated using a Biacore T100 (GE Healthcare, Uppsala, Sweden). Experiments were performed at 25 °C and data were fitted with a 1:1 Langmuir interaction model. HER2 ectodomain-Fc (96 kDa) was diluted to 50 µg/mL in sodium acetate buffer, pH 5.0, and immobilized by amine-coupling on a CM5 chip (GE Healthcare, Uppsala, Sweden) at 1270 RU. VHHs were diluted in 20 mM Hepes, 150 mM NaCl, 3 mM EDTA, 0.005% Tween 20, pH 7.4 and injected as analytes at 30 µL/min at 7 concentrations between 250 and 3.5 nM. The kinetics were collected in a unique sequence of injections and surface regeneration (2 mM NaOH for 8 s at 30 µL/min) took place only between two successive series of measurements.

### 2.4. Enzyme-Linked Immunosorbent Assay (ELISA)

One hundred μL of VHH-GFP (200 nM) were immobilized overnight at 4 °C in plate wells using carbonate buffer, pH 9.2. After washing in PBS plus 0.05% Tween 20, samples were coated 1 h with blocking solution (PBS, 5% milk, 0.05% Tween 20). One hundred μL of anti-GFP, mCherry-fused VHH at two concentrations (200 nM and 1 μM) were resuspended in PBS, 1% milk, 0.05% Tween 20 and incubated for 2 h. After 4 washes in PBS, 0.05% Tween 20, the plate was analyzed using a plate reader (Ex: 580 nm, Em: 610 nm).

### 2.5. Homology Modeling

Template structure PDB ID 3TPK [18] was identified by searching the protein data bank for VHH with frameworks with sequence identity >80% and E-Value Cutoff 10.0., as in Ref. [19]. Specific mutations and insertions were done manually with DeepView-Swiss-PdbViewer 4.1 [20] to obtain selected VHHs starting models that underwent the MD protocol reported below.

### 2.6. Molecular Dynamics Simulations

Each VHH was minimized, placed in a cubic box with a water layer of 0.7 nm and underwent a second minimization. We used AMBER99SB-ILDN [21] force field and Simple Point Charge water before performing NVT and NPT equilibrations for 100 ps, followed by 500 ns NPT production run at 350 K. The iteration time step was set to 2 fs with the Verlet integrator and LINCS [22] constraint. The Particle Mesh Ewald summation accounted for long range electrostatic interactions. The temperature was controlled with a modified Berendsen thermostat [23], the pressure with an isotropic Parrinello–Rahman at 1 bar. Configurations and energies were sampled every 0.5 ns. All the simulations and their analysis were run as implemented in the GROMACS package [24].

### 2.7. Clustering

We clustered 800 structures extracted from the last 400 ns of the molecular dynamics simulations using the g_cluster program of GROMACS [24]. We employed the gromos method [25] for clustering the structures, selecting as root-mean-square deviation (RMSD) cut-off value the RMSD average value obtained from the matrix of structure combinations. We considered only the C-alpha structure of the protein for the least squares fit and RMSD calculation in the clustering calculation. For each VHH, the cluster analysis led to 3–5 clusters with the most populated one accounting for the 80–90% of the sampled configurations. One reference conformation for each VHH was then chosen for further analysis.

### 2.8. Solubility Prediction

We calculate the CamSol structurally corrected solubility of the reference VHH conformations by using the webserver tool [26], and applying a patch radius of 10 Å, which defines the maximum distance of interaction between residues in the 3D space, and pH = 7. The putative aggregation hotspots were defined by those residues exposed to the solvent within a distance of 6 Å of the sidechain of the poorest solubility residues, according to the CamSol score. Then, the aggregation propensity of each hotspot is estimated as the sum of solubility scores of all residues belonging to the same hotspot.

### 2.9. Docking Calculations

VHHs were docked to selected VHH epitopes with the web “easy interface” of HADDOCK [27] and its standard parameters. Active residues of the binding sites were defined for each reference VHH conformation (see Section 2.7) following the protocol developed in Ref. [10]. In short, we used two complementary approaches to define the active residues of the potential aggregation hotspots. First, we used the webtool Interprosurf [28] to analyze the hydrophobic solvent-accessible residues of each VHH. Second, we evaluated the structural differences between the mutants and the wild-type by calculating the difference between their contact maps. A projection of the difference matrix over each residue is used to identify the most affected residues by the mutations. Finally, we identified the residues selected from both approaches that shape binding surfaces and pockets defining possible aggregation hotspots. Passive residues in each binding site were automatically defined by HADDOCK.

## 3. Results and Discussion

**Figure 1:**
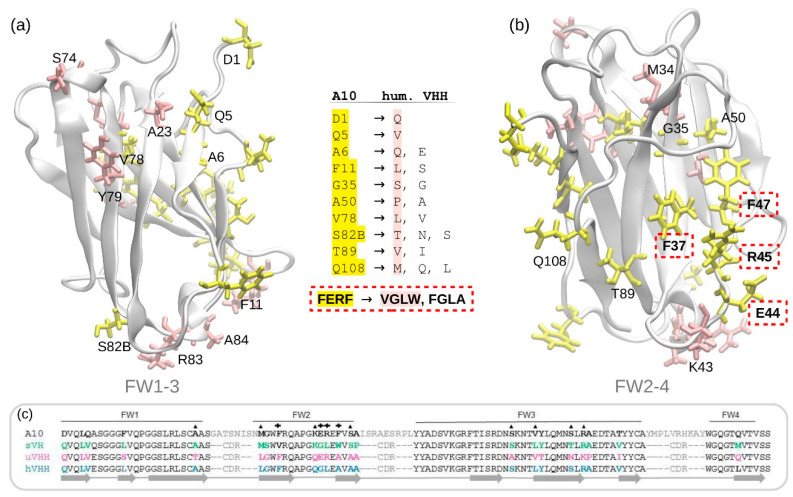
Structural analysis of the framework of VHH A10. Potential camelid residues to be mutated (yellow) and already humanized residues (pink) in the sites formed by the (**a**) framework 1-3 (FW1-3), and (**b**) framework 2-4 (FW2-4). In the center, camelid residues of A10 are compared with the model VH residues [29,30,31]. The key hallmarks FERF are highlighted by a red rectangle. (**c**) Sequence alignment of the frameworks of A10, the reference human VH (sVH) [29], a universal VHH framework (uVHH) [30], and the most humanized VHH sequence available (hVHH) [9]. Crosses indicate the FERF/VGLW hallmarks, while triangles show the already humanized residues of A10.

The nanobody A10 was selected by panning a synthetic library and its *K*D for its antigen, the extracellular domain of HER2, was 4 nM [3,16]. Such VHH was expressed fused to different tags with yields in the range of 3–20 mg/L culture medium and was suitable for HER2 detection both in vitro and in vivo [3]. The library to which A10 belongs has been built by inserting hypermutated CDRs into a fixed framework (FW) that was selected identifying “human-like” residues among known llama variants (Figure 1a,b). Isolated clones can differ for single mutations inserted during the amplification cycles of the genetic material. In the specific case of A10, the camelid hallmark residues in the FW2 are 37F, 44E, 45R, and 47F (boxed in Figure 1b and indicated with + and identified according to conventional numbering in Figure 1c). We selected A10 as a candidate for testing different approaches aimed at VHH humanization with the perspective of its employment in in vivo applications. Specifically, we compared alternative strategies to mutate the camelid conserved residues into their humanized version, with a particular interest for the signature (FERF versus VGLW) that distinguish camelid and conventional heavy-chain variable regions.

### 3.1. A10 Humanization by Framework Mutations

The first considered approach was empirical, based on the sequence comparison of A10 (synthetic library framework) with the sequences of: (i) a stable human VH domain used for library construction [29]; (ii) a universal VHH framework suitable for CDR grafting [30]; (iii) the most humanized VHH sequence available which still preserved sufficient stability [9]. Given the similarity between *Camelidae* VHH and human VH3 sequences, the DP47-VH sequence [31] was consulted as a further reference. The sequence alignment (Figure 1c) enabled the identification of several residues potentially interesting as mutation candidates (in yellow in the structures of Figure 1a,b), whereas confirmed that some A10 residues were already humanized (in pink in the structures of Figure 1a,b and marked with top triangles in the sequence of Figure 1c). Accordingly, three mutants were tested, the sequences of which are reported in Figure S1. Mutant 1 was humanized in all the identified critical residues, in mutant 2 some not-key residues were preserved in the original (llama) form with the idea that this intermediate condition between the original and the humanized version could result in a more stable structure. In mutant 3, A10 CDRs were grafted into the universal VHH sequence [30] humanized at the two FW2 hallmarks 44 and 45 (ER to GL). Such mutants yielded only soluble aggregates and, consequently, were dismissed without further characterization while we looked for alternative strategies.

### 3.2. Anti-FGFR1 C8: An Unusual Human-Like Nanobody

**Figure 2:**
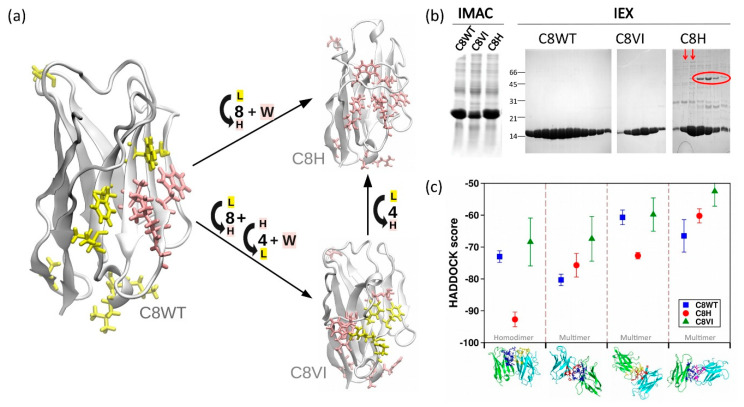
Expression of anti-EFGR C8WT and mutants. (**a**) Structural description of the mutating strategy to transform C8WT into C8VI and C8H. Human residues are colored in pink while llama residues are yellow. (**b**) IMAC and IEX results obtained during the expression of the three VHHs. Red arrows indicate the low propensity of C8H to form multimers. (**c**) Docking prediction of the propensity of VHHs to form multimers. Higher score values means lower predicted aggregation.

In the past, we isolated from a native llama library an anti-FGFR1 nanobody (C8WT), with affinity in the low nanomolar range for its antigen that had the human hallmarks (VGLW) and despite this characteristic was expressed at high yields as a stable recombinant protein [1]. We wished to evaluate the capacity of this scaffold to adsorb mutations without losing its stability. Its sequence was engineered to obtain two mutants with opposite features (Figure 2a). In the first case (C8H), the original sequence was further humanized by mutating those FW residues that, according to the humanized model sequences summarized in Figure 1, still differed with respect to the human VH (Figure S2a). In the second (C8VI), only the human hallmarks VGLW of C8H were mutated into their camelid counterparts, whereas all the other residues maintained the human-like fingerprint. C8H and C8VI can be interconverted into each other by mutating the hallmark VGLW into FERF and vice versa. The mutants were expressed and purified together with the wild-type construct by IMAC and IEX (Figure 2b). The yield of the totally humanized mutant C8H (3.1 mg/L culture) was less than half of the yields obtained with C8WT (7.5 mg/L) and one third of the protein produced using the camelized mutant C8VI (9.2 mg/L).

In a previous work, the HADDOCK software demonstrated being a robust descriptor to predict the usable yields of VHHs [10] and was therefore applied to the C8WT constructs to evaluate their theoretical propensity to form multimers that can favor colloidal aggregation. Apart from a single conformation of C8H, which can lead to the formation of a stable homodimer organized as in an Fv where one VHH takes the structural place of the variable domain of the light chain (VL), the HADDOCK scores indicated that the constructs had low dimerization propensity (high HADDOCK score values), with C8VI that performed always better than the others (Figure 2c). These theoretical predictions found support in the C8H IEX-purified fractions analyzed by SDS-PAGE. Despite the denaturing conditions, larger contaminants with molecular weight compatible with dimers and tetramers were observed (circled in Figure 2b). The nanoDSF data indicate that the thermal stability of the mutants decreased (Figure S2b). Nevertheless, the homogeneous monomeric fractions of all constructs remained apparently stable when incubated for 15 days at −20 °C, 4 °C or 21 °C. Such experimental result suggests that C8H intermediates can be trapped into aggregation-prone conformations during folding, resulting in lower yields of soluble protein. In contrast, the protein fraction that reached its native structure shows negligible propensity to form large multimers (indicated by red arrows in Figure 2b). It was not taken for granted that the C8WT scaffold could stand multiple mutations towards both the human and the camelid sequences without compromising the single-domain stability because of the multiple interactions among residues belonging to different secondary structure elements that normally contribute to single-domain stabilization [8]. Regretfully, it is difficult to rationalize the robustness of the C8WT scaffold in the absence of a crystallographic structure that could conclusively clarify at the molecular level the stabilizing effects of intermolecular interactions. Therefore, we moved further to evaluate the stability features of C8WT by carrying out a new strategy for the humanization of A10, based on the grafting of A10 CDRs onto C8WT scaffold. Additionally, our approach unveiled the role of critical residues and CDR orientation in the peculiar stability of C8WT.

### 3.3. A10 Humanization by Grafting onto C8WT

When A10 and C8WT sequences are compared, it appears that A10 FW differs from C8WT by 14 amino acids and possesses a further residue (Trp) at the beginning of FW4 (Figure S3a). In A10 seven of the modified residues have “human fingerprint”, the other seven are typical from llama sequences. All these residues have their side chains exposed to the solvent, except for the amino acid at position 78 (Val in A10 and Leu in C8WT), whose side chain is located between the beta-sheets. Eight out of 14 residues are located on the same VHH surface composed by beta-sheets of the FW2 and FW4, whereas the other residues are spread over the beta-sheets of the FW1 and present in different coils (see Figure S3). The surface formed by the FW2 and FW4 corresponds to that interacting with the VL domain in conventional antibodies and, consequently, the region that during evolution underwent more modifications to adapt to the heavy-chain only structure of camelid antibodies. Surprisingly, C8WT, which was isolated from a naïve llama library (Figure S3b), possesses human VGLW hallmarks in FW 2 instead of the camelid hallmarks (FERF in A10). In contrast, its residues Tyr35, Ser50, Leu89, and Phe91 are typical of llama sequences. A10, recovered from a synthetic library designed to favor the most human options among the available llama FW sequences (except for the hallmarks) [12,32], showed humanized residues at three of these sites (Gly35, Ala50, and Tyr91).

**Figure 3:**
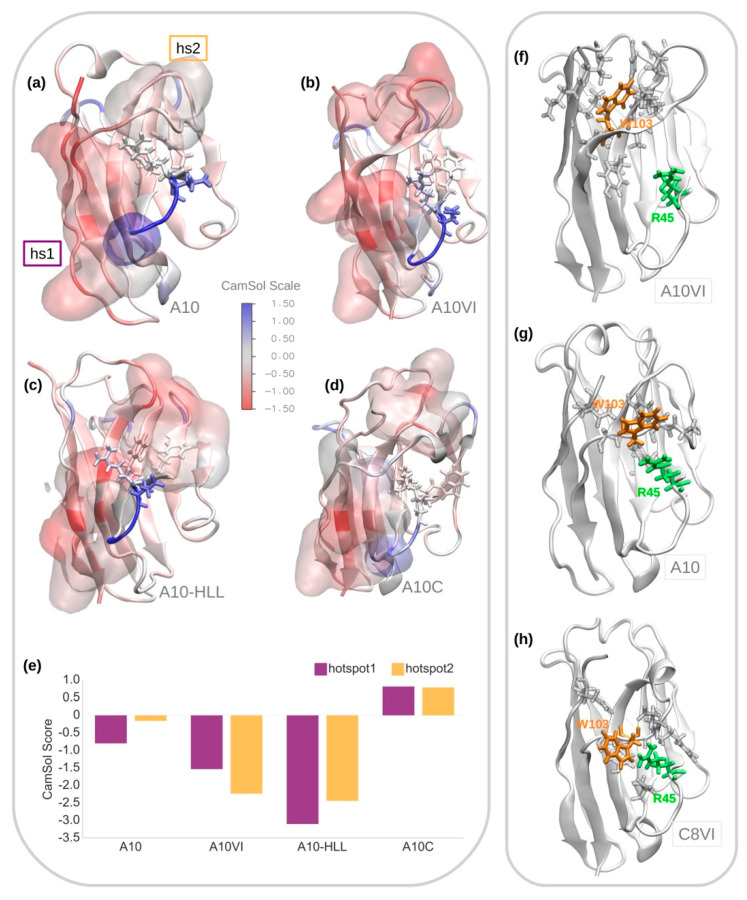
Structural analysis of A10 and its mutants. Representative MD conformations of (**a**) A10, (**b**) A10VI, (**c**) A10-HLL and (**d**) A10C colored according to the solubility CamSol residue scores. Solubility hotspots are represented as surfaces while the FW2 hallmarks VGLW/FERF are showed in licorice. (**e**) Solubility CamSol scores of the two hotspots identified in the structures of all 4 VHHs. Lower scores values (higher degree of red in the scale) indicate regions with poor solubility or higher aggregation propensity. Structural comparison of the interaction between Trp103 (orange) and Arg45 (green) in the most representative conformations of (**f**) A10VI, (**g**) A10, and (**h**) C8VI. All residues located within a distance of 4 Å of Trp103 are also indicated.

We analyzed a set of VHH mutants with intermediate sequences between those of A10 and of A10 grafted into C8WT (A10C). These were expressed in bacteria and, in parallel, we performed an in silico analysis of the solubility/aggregation propensity of the two most representative among them (Figure 3). One of them corresponds to the framework of C8VI and the other framework sequence is an intermediate between C8VI and C8WT (Figure S4).

A10VI, the VHH resulting from grafting the A10 into the C8VI framework, differs from A10 in only 4 residues (Leu11 in FW1, Ser35 in FW2, the internally oriented residue Leu78, and Val89 in FW3, Figure S4) and for the deletion of the residue Tyr102, which usually is considered to belong to the CDR3, but is absent in C8WT. In A10-HLL, the human hallmarks VGLW were mutated in the camelid counterparts, while the llama residues Tyr35, Ser50, Leu89, Phe91 were humanized inside the framework of A10C. Altogether, A10-HLL and A10C differ therefore by 8 residues, while A10-HLL and A10VI differ by 4 residues (Figure S4). The contribution in terms of solubility provided by each of the residues exposed in the most representative conformations suitable for all the four VHHs was computed by using the CamSol tool [26] (Figure 3a–d). Also, the aggregation propensity of the identified hotspots was estimated as a sum of the solubility scores of the residues that constitute each hotspot (Figure 3e). In A10, the residue solubility scores indicated the existence of one single aggregation hotspot, called hotspot1 (Figure 3e), formed mainly by the residues located in both C- and N-terminal ends (Figure 3a). In this VHH, the predicted conformation of CDR3 hides the hydrophobic residues present on the surface constituted by FW2-4, avoiding their exposure towards the solvent and thus preventing the formation of a further aggregation hotspot, as confirmed by the hotspot score (Figure 3e). The protective CDR3 screening effect is lost in A10VI and A10-HLL, with the consequent solvent exposure of the hydrophobic residues of the FW2-4 surface (Figure 3b,c). In A10VI, the presence of Trp103, which is absent in A10C, might result in an alternative folding of the A10 CDR3 loop that exposes part of its hydrophobic residues and creates an additional hotspot, i.e., hotspot2 (Figure 3f). More specifically, the model in A10 indicates that Trp103 interacts with Arg45, stabilizing the protective CDR3 conformation (Figure 3g), while in A10VI the same residue remains between the CDR1 and CDR3 loops, interacting with amino acids of the N-term tail (Figure 3f). It should be noted that in our simulations we found Trp103 in C8VI in a similar stable conformation as the one observed in A10, in which it is interacting with Arg45 (Figure 3h). In parallel, the mutation Thr89 to Leu89, which is included in the hotspot1, enhances its aggregation propensity (Figure 3b). The loss of CDR3-dependent stabilizing effect is even clearer in the case of A10-HLL. Here the CDR3 does not interact with the FW2-4 surface (Figure 3c) since the presence of Arg45, a bulky and charged residue, hinders the protective interaction between CDR3 and FW2 residues. The absence of Trp103 in A10-HLL might enhance the hindering effect of Arg45. As a result, two new evident aggregation hotspots appear in the intermediate mutants A10VI and A10-HLL (Figure 3e).

The unexpected high solubility of A10C, as obtained by the experimental results, seems due to the sum of more factors. The removal of Trp103, in combination with the presence of the hydrophobic residues VGLW in FW2 and the absence of Arg45, apparently allowed the protective H3 loop repositioning over the FW2 area. This conformation screens the hydrophobic residues of both CDR3 and FW2 (Figure 3d). Moreover, there is a conformation rearrangement with a partial twist of FW2 beta-sheets by which the hallmark residues Val37 and Leu45 are stabilized by their reciprocal interaction. Consequently, the aggregation propensity of the defined hotspots is predicted to be negligible.

The mutants designed for the in silico analysis were expressed and purified by IMAC and size exclusion chromatography (SEC) (Table 1). The SEC profiles were also used to identify the presence of aggregates (Figure S5).

**Figure 4:**
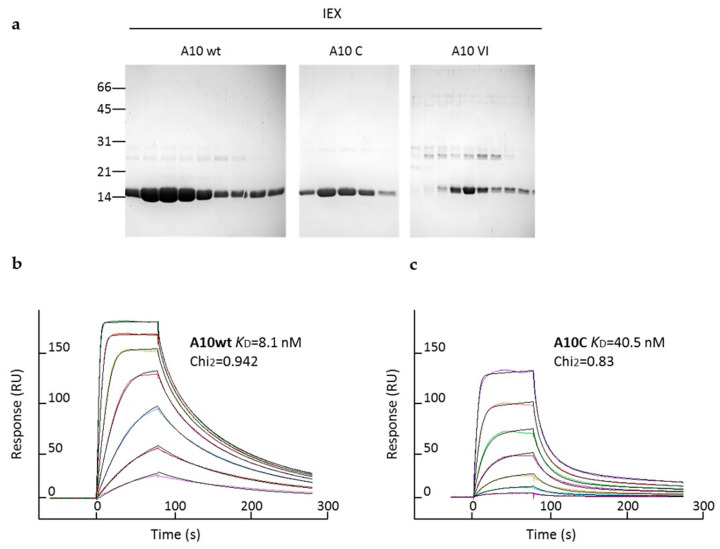
Evaluation of A10/C8WT chimeras by (**a**) IEX and (**b**,**c**) SPR. The IEX elution profiles (**a**) show the negligible propensity of A10C to dimerize. Such construct preserved the binding affinity for HER2 (**c**).

The production experiments confirmed the in silico analyses. The mutants A10VI and A10-HLL produced unstable proteins, whereas the A10C chimera yields were discrete (4.9 mg/L culture), the protein appeared monodispersed (Figure 4a) and preserved the binding specificity of A10 for HER2 (Figure 4b), despite its five-fold drop in affinity (Figure 4c). Since we cannot rule out that further residues outside the CDRs contribute to the paratope and therefore to the antigen binding, the measured *K*_D_ seems to strongly support the hypothesis that the grafted CDRs could arrange into a highly functional structure inside the C8WT scaffold. In conclusion, the experimental data confirmed the possibility of grafting A10 CDRs in the “naturally human” C8WT scaffold. Despite the frameworks of A10 and C8VI differ by only 4 residues, the grafting of A10 CDRs resulted in important lower yields, in agreement with the computational analysis, which indicated that the stabilizing effect of original CDRs present in C8VI was lost in the new mutant.

**Figure 5:**
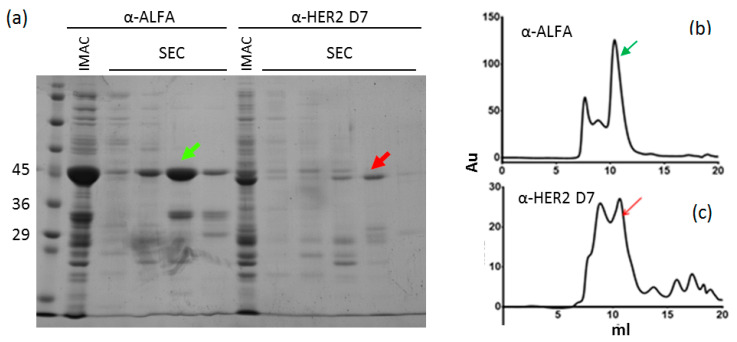
Evaluation of chimeras prepared by CDR grafting into C8WT frameworks. Both the anti-ALFA tag and the anti-HER2 chimera fusion-nanobodies (VHH-mClover) were purified as a major band after IMAC (**a**) and the major elution peak in SEC (indicated by colored arrows) corresponded to their monomeric construct (**b**,**c**).

In parallel with the theoretical simulations and the characterization of the A10/C8WT mutants corresponding to VHHs with hybrid human/llama sequence, we tested the suitability of C8WT to act as a universal acceptor of VHH CDRs to transform camelid single domains into stable human-like VHs. The sequences corresponding to the CDRs of four VHHs (anti-GFP, anti-ALFA tag, and two anti-HER2, Figure S6) were cloned into the C8WT scaffold and the constructs were expressed as fusions with a fluorescent protein (mCherry for the anti-GFP, mClover for the others). All constructs were produced as soluble proteins and initially purified by metal-affinity chromatography (IMAC). The immunofluorescent fusion proteins represented the major band in the SDS-PAGE (Figure 5a and Figure S7). The samples underwent gel filtration (SEC) to evaluate whether the chimeras were monomers and indeed their major fraction eluted at the expected retention time. As visible in the SDS-PAGE of Figure 5b, the yields of the different clones differ significantly and were evaluated ranging from 0.5 mg/L to some mg/L. The anti-GFP clone fused to mCherry was the only one to produce both a monomer and a dimer (Figure S7), despite mCherry should not be prone to dimerization [33]. The dimer fraction was run again by SEC and eluted as a single peak. Such clone retained at least part of its specific binding for its antigen since was successfully used to detect GFP-fused proteins by ELISA test (Figure S8). Given the multiple interactions existing between different FW and CDR residues that control VHH stability and functionality, it is improbable that these properties are totally preserved after CDR grafting in any scaffold, but our results indicated that C8WT is stable enough to adapt to a variety of CDR sequences and enable the production of stable chimeras and that at least some of them are still functional.

## 4. Conclusions

The process of progressive humanization of camelid sequences seems to find its limits when the VHH hallmarks present in FW2 must be converted into their human counterparts, with consequent loss of stability and increased propensity to aggregate [8,34]. Our results demonstrated that it is feasible to obtain soluble humanized chimeras by exploiting the nanobody C8WT scaffold, which naturally harbors human hallmarks in its sequence, to graft the CDRs of donor nanobodies.

In both tested cases (C8WT grafted with CDRs of anti-HER2 and anti-GFP nanobodies), the chimera still preserved their binding capacity for the corresponding antigens. These results overcame our expectations because the in silico analyses clearly showed how the C8WT stability is the result of a specific interaction between FW2 and CDR3 residues and that such equilibrium could be challenged by the modification of a single residue in FW4. However, the CDR3 bending over FW2 has been described repeatedly also for (canonical) camelid VHHs [14,35,36,37] and might be a relatively frequent event in this class of binders. Nevertheless, recent systematic analyses of nanobody structures have shown two critical aspects of their paratopes [32,38,39,40]. First, the dimension of all three CDRs can largely vary and include a different number of adjacent residues. Second, the contribution of FW residues to the paratope is relatively common and in other cases FW amino acids may play an indirect but critical role by imposing structural constraints to the loops involved in the antigen binding.

Altogether, such pieces of information suggest that grafting “standard sequences” on a putative “universal” scaffold could often lead to deceiving results. Consequently, we do not believe that C8WT, as any other single-domain VHH, can represent a universal CDR acceptor, even though its scaffold might represent a valid opportunity for the humanization of single nanobodies, as indicated by the examples reported in this work. In contrast, we consider C8WT more suitable as the scaffold for preparing synthetic libraries or starting the in silico development of new binders [41]. Of course, many combinations will be not functional, but this is probably true for any synthetic library and unsuitable clones will be eliminated during panning, whereas the advantage of such collection will be that the final isolated binders, selected according to their functionality and stability, will already possess a human-like sequence that will simplify their use as reagents for in vivo clinical applications.

We wish to underline that this work shows as well how the computational tools have become not only reliable instruments to integrate the experimental data but to provide fast guidance and valuable insights relative to structural information.

## Figures and Tables

**Table 1:** Characterization of A10 mutants: A10 wt. and its mutants were purified first by IMAC and then by SEC and the protein yields were calculated after each step. Only the proteins eluted as a monomer were considered after SEC.

Constructs	Yield IMAC (mg/L)	Yield SEC (mg/L)	Aggregation State (According to SEC Profile)
A10	12.6	8.9	monodispersed
A10-HLL	2.0	0	polydispersed
A10C	7.6	4.9	monodispersed
A10VI	4.5	0.6	polydispersed

## Supplementary Materials

The following are available online at https://www.mdpi.com/2218-273X/11/2/163/s1, Figure S1: Sequence-derived mutants of A10. Figure S2: Sequences of C8WT and its mutants. Figure S3: Structural comparison between A10 and C8WT. Figure S4: Structures and sequences of A10 and their mutants A10VI, A10HLL, and A10C. Figure S5: Elution profiles after gel filtration. Figure S6: Grafting nanobody CDRs into C8WT scaffold. Figure S7: C8WT chimera expression and monomeric behavior. Figure S8: Specific antigen recognition of the C8-αGFP chimera.
