# Supplementary material for: Effect of Humanizing Mutations on the Stability of the Llama Single-Domain Variable Region
Source: Biomolecules. 2021 Jan 26;11(2):163. doi: 10.3390/biom11020163 (PMC7911018; doi:10.3390/biom11020163)
Supplement: Supplementary file 1 [file biomolecules-11-00163-s001.pdf]

Supplementary Material

Effect of humanizing mutations on the stability of the llama single-domain variable region

Miguel A. Soler[1], Barbara Medagli[2], Jiewen Wang[3], Sandra Oloketuyi[4], Gregor Bajc[5], He Huang[3], Sara Fortuna[2], Ario de Marco[4]

[1] Italian Institute of Technology (IIT), Via Melen – 83, B Block, 16152 - Genova, Italy; miguel.soler@iit.it

[2] Department of Chemical and Pharmaceutical Sciences, University of Trieste, Via L. Giorgieri 1, 34127 - Trieste, Italy; bmedagli@units.it; s.fortuna@units.it

[3] Key Laboratory of Systems Bioengineering (Ministry of Education), School of Chemical Engineering & Technology, Tianjin University, Tianjin 300072, China; wangjw@tju.edu.cn ; huang@tju.edu.cn

[4] Lab of Environmental and Life Sciences, University of Nova Gorica, Vipavska cesta 13, 5000, Rožna Dolina - Nova Gorica, Slovenia ; sandra.folarin.oloketuyi@ung.si ; ario.demarco@ung.si

[5] Department of Biology, Biotechnical Faculty, University of Ljubljana, Večna pot 111, 1000 Ljubljana, Slovenia; gregor.bajc@bf.uni-lj.si

*** Correspondence: Ario de Marco, Lab of Environmental and Life Sciences, University of Nova Gorica, Vipavska cesta 13, 5000, Rožna Dolina-Nova Gorica, Slovenia. ario.demarco@ung.si, Phone: +386 (05) 3315295, Fax: +386 (05) 90 99 722; Miguel A. Soler, Italian Institute of Technology (IIT), Via Melen – 83, B Block, 16152 - Genova, Italy; miguel.soler@iit.it**

```
A10   DVQLQASGGGFVQPGGSLRLSCAASGATSNISNMGWFRQAPGKEREFVSAISRAESRPLYYADSVKGRFTISRDNSKNTVYLQMNSLRAEDTATYYCAYMPLVRHKAYWGQGTQVTVSS
Mut1  QVQLVESGGGLVQPGGSLRLSCAASGATSNISNMSWFRQAPGKGLEWVSAISRAESRPLYYADSVKGRFTISRDNSKNTLYLQMNSLRAEDTAVYYCAYMPLVRHKAYWGQGTLVTVSS
Mut2  DVQLVESGGGLVQPGGSLRLSCAASGATSNISNMSWFRQAPGKGLEWVSAISRAESRPLYYADSVKGRFTISRDNSKNTLYLQMNSLRAEDTATYYCAYMPLVRHKAYWGQGTQVTVSS
Mut3  QVQLVESGGGSVQPGGSLRLSCTASGATSNISNLGWFRQAPGQGLEAVAAISRAESRPLYYADSVKGRFTISRDNAKNTVTLQMNNLKPEDTAIYYCAYMPLVRHKAYWGQGTQVTVSS
```

Green: human VH [29]
Magenta: universal VHH [30]
Blue: humanized VHH [9]

Figure S1. Sequence-derived mutants. A10 camelid hallmarks were humanized in combination with mutations involving further residues participating to either human or camelid fingerprints. Color code hVH: human VH (green), uVHH: universal VHH (magenta), hVHH: humanized VHH (blue).

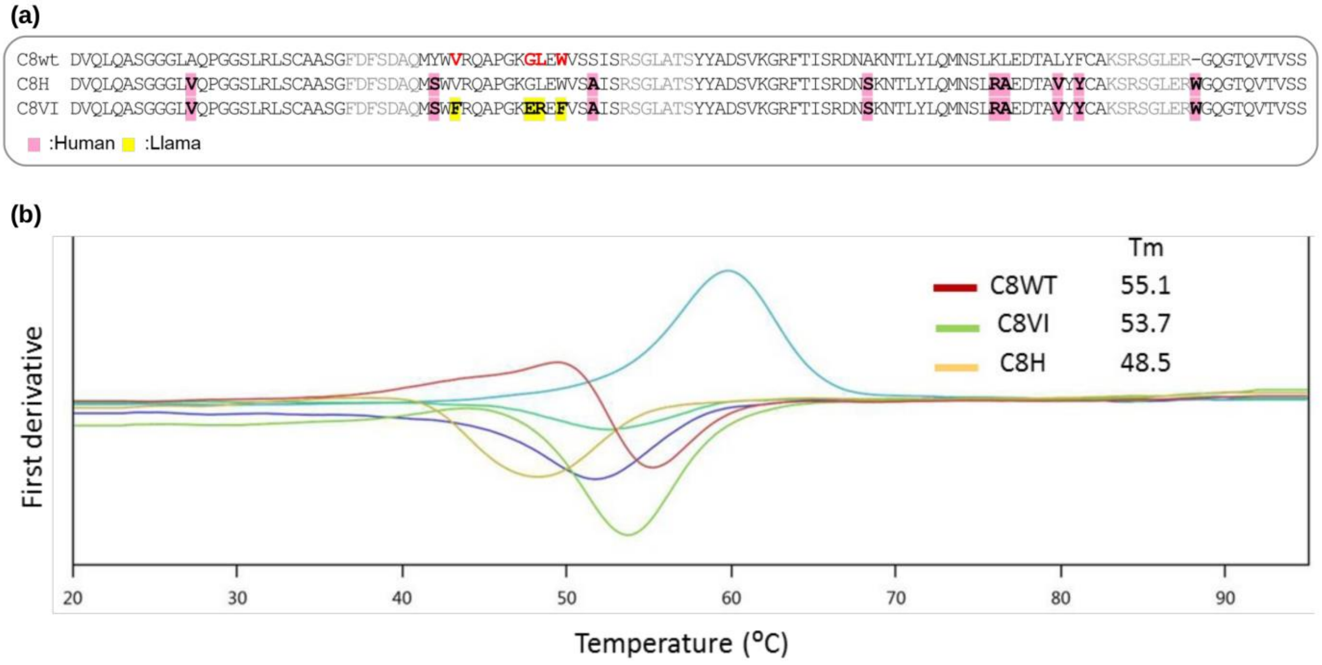

Figure S2. Sequences (a) and thermal stability (b) of C8 and its mutants. (a) The naturally present "human hallmarks" of the llama VHH C8 are in red. The residues modified in the mutants (C8H: C8 further humanized, C8VI: C8H camelized) are colored in yellow and pink. (b) The first derivative functions and corresponding Tm values calculated by DSF are reported. The blue/violet curves refer to control Nbs

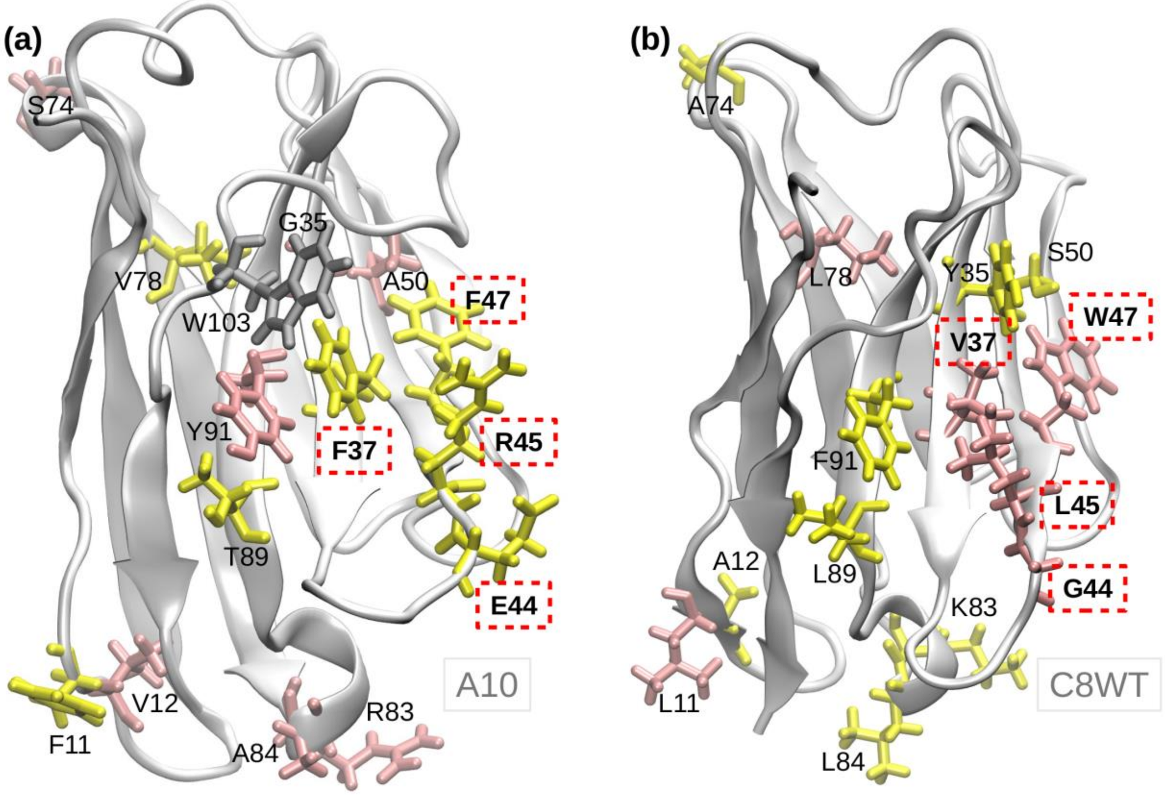

Figure S3. Structural comparison between (a) A10 and (b) C8WT. Humanized residues are colored in pink while camelid residues are yellow. The FW2 hallmarks FERF/VGLW are highlighted by a red rectangle. A10 further differs from C8WT by the insertion of Trp103 (gray).

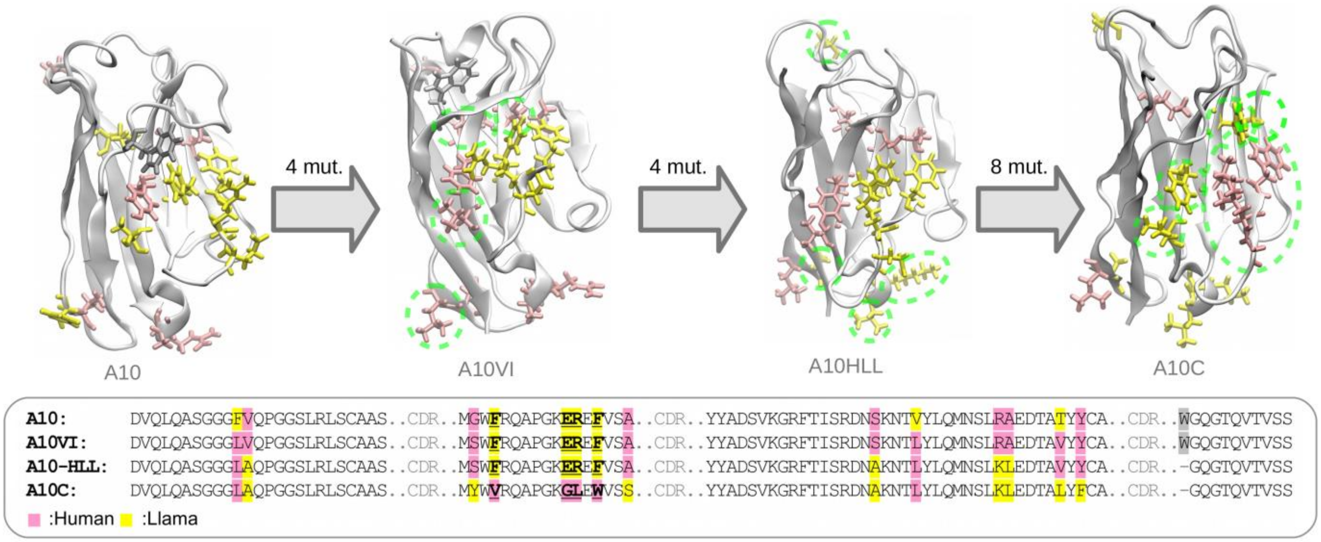

Figure S4. Structures and sequences of A10 and their mutants A10VI, A10-HLL, and A10C. Residues mutated at each step are highlighted with green dashed circle in each structure. FERF/VGLW signatures are underlined.

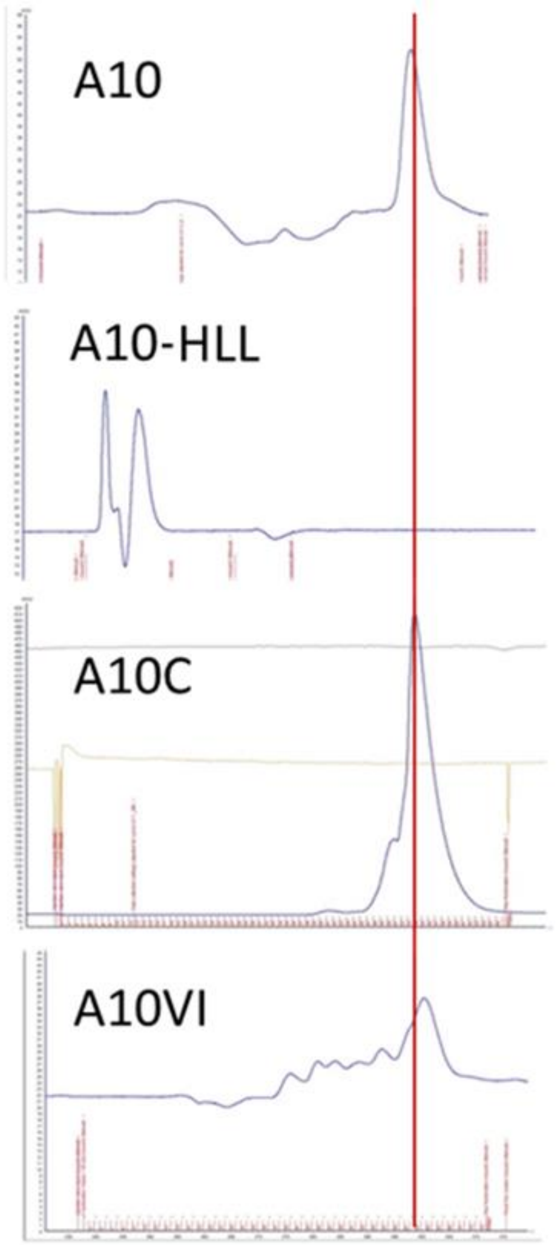

Figure S5. Elution profiles of A10 and A10 mutants after gel filtration.

```
C8wt     DVQLQASGGGLAQPGGSLRLSCAASGFDFSDAQ---MYWVRQAPGKGLEWVSSISRSGLATSYYADSVKGRFTISRDNAKNTLYLQMNSLKLEDTALYFCAKSRS----GLERGQGTQVTVSS
C8_GFP   DVQLQASGGGLAQPGGSLRLSCAASGFPVNRYS---MYWVRQAPGKGLEWVSSISSAGDRSSYYADSVKGRFTISRDNAKNTLYLQMNSLKLEDTALYFCAVN------VGFEGQGTQVTVSS
C8_A2    DVQLQASGGGLAQPGGSLRLSCAASGTPSTAWGN--MYWVRQAPGKGLEWVSSISYGDGKVLYYADSVKGRFTISRDNAKNTLYLQMNSLKLEDTALYFCANLPY---KRKQMGQGTQVTVSS
C8_D7    DVQLQASGGGLAQPGGSLRLSCAASGDSSRHSG---MYWVRQAPGKGLEWVSSISFRGNFESYYADSVKGRFTISRDNAKNTLYLQMNSLKLEDTALYFCAELPLGSRGSRQSGQGTQVTVSS
C8_ALFA DVQLQASGGGLAQPGGSLRLSCAASGVTISALNAMAMYWVRQAPGKGLEWVSSISERGN-AMYYADSVKGRFTISRDNAKNTLYLQMNSLKLEDTALYFCAVLEDRVDSFHDYGQGTQVTVSS
```

Figure S6. Grafting nanobody CDRs into C8 scaffold. The CDR sequences of four different functional nanobodies (in red) were inserted into the C8 framework.

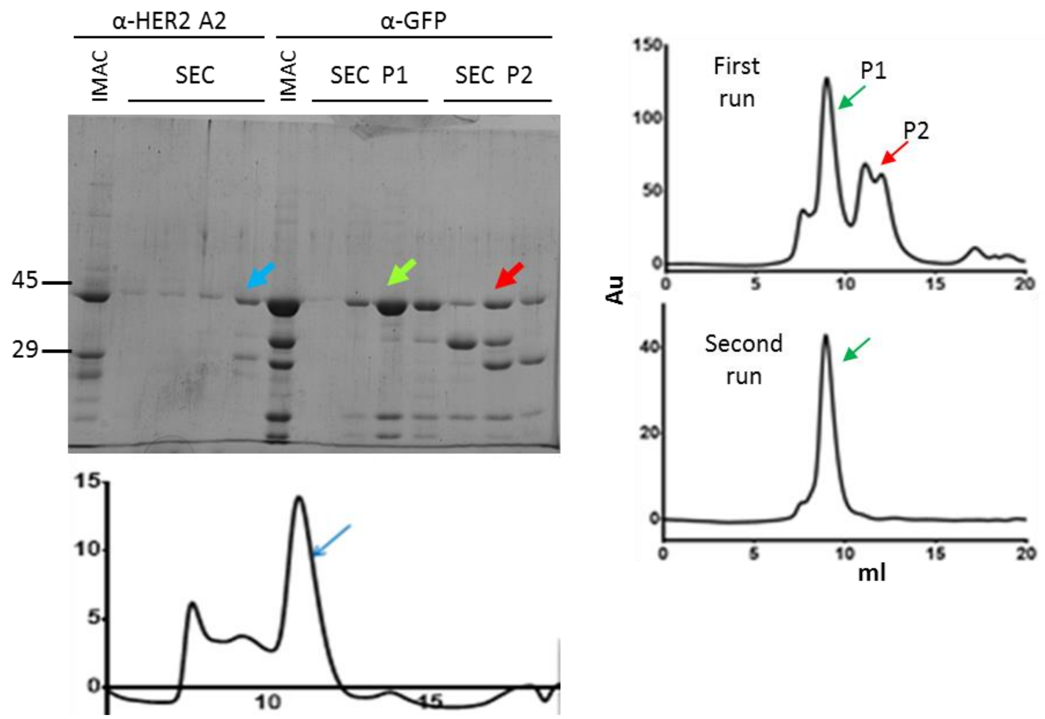

Figure S7. C8 chimera expression and monomeric behavior. Nanobody chimeras of C8 scaffold and CDRs from anti-HER2 (A2) and anti-GFP were purified by IMAC and SEC. The proteins present in SEC peaks were separated by SDS-PAGE ; in the case of anti-GFP, both peak1 (P1 : dimer) and peak2 (P2 : monomer plus degradation products) were run in parallel. The anti-GFP P1 (green) was run again (second run) and eluted at the same retention volume.

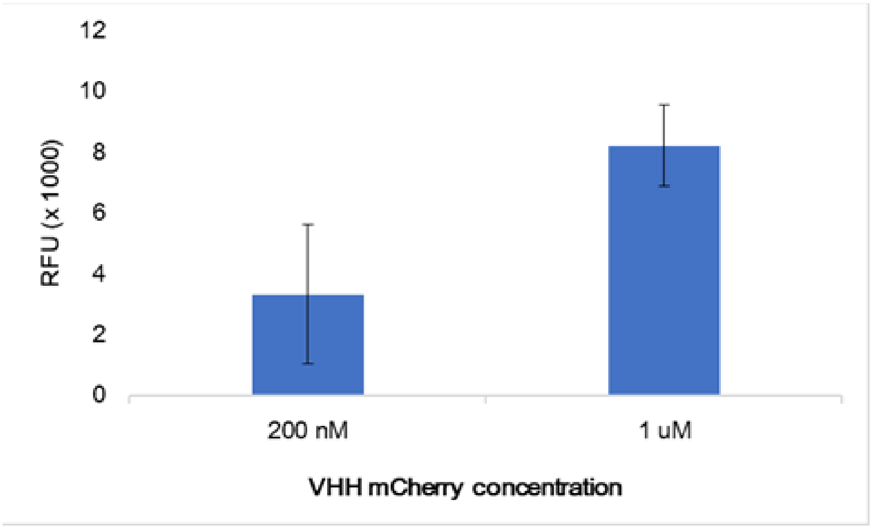

Figure S8. Specific antigen recognition of the C8-αGFP chimera. The chimera formed by C8 scaffold and anti-GFP CDRs was used to evaluate its capacity of specifically binding to its antigen in a sandwich ELISA test. Histograms obtained by averaging 2 measures and error bars calculated as (max value – min value)/2.
